# Supplementary material for: Evaluating metabarcoding to analyse diet composition of species foraging in anthropogenic landscapes using Ion Torrent and Illumina sequencing
Source: Sci Rep. 2018 Nov 20;8:17091. doi: 10.1038/s41598-018-34430-7 (PMC6244006; doi:10.1038/s41598-018-34430-7)
Supplement: Supplementary file 1 — Supplementary Tables [file 41598_2018_34430_MOESM1_ESM.pdf]

Evaluating metabarcoding to analyse diet composition of species foraging in anthropogenic landscapes using Ion Torrent and Illumina sequencing

M.-A. Forin-Wiart, M.-L. Poulle, S. Piry, F. Cosson, C. Larose and M. Galan.

Supplementary Table S1: Details on the calibration study

Detailed composition of the daily rations distributed during two sessions to the housebound cat, the collected faecal samples and food item detection [not detected (-) or one replicate (one) or two replicates (two)] per food category and HTS platform (Ion PGM and MiSeq runs).

Sessions	Days	Daily composition			Faecal samples	DNA metabarcoding detections					
						Prey items		Pet food		Surplus meat/fish	
		Prey items	Pet food	Surplus meat/fish		Ion PGM	MiSeq	Ion PGM	MiSeq	Ion PGM	MiSeq
	1		x								
	2		x								
	3	x	x								
	4	x	x		1	-	-	-	two		
	5	x	x		2	two	two	-	-		
	6	x	x								
	7	x	x		3	two	two	-	-		
	8	x			4	two	two	-	-		
	9		x		5	one	two	-	-		
	10		x								
1	11		x		6	-	two	-	one		
	12		x								
	13		x		7			one	two		
	14		x		8			one	two		
	15-1		x		9			-	two		
	15-2		x		10			one	two		
	16		x		11			one	-		
	17		x		12			-	two		
	18		x		13			two	one		
	19		x		14			-	one		
	20		x		15			-	two		
	21		x		16			one	one		
	22		x		17			-	-		

Supplementary Table S1 (Continued)

Sessions	Days	Daily composition			Faecal samples	DNA metabarcoding detections					
						Prey items		Pet food		Surplus meat/fish	
		Prey items	Pet food	Surplus meat/fish		Ion PGM	MiSeq	Ion PGM	MiSeq	Ion PGM	MiSeq
	1		x								
	2		x	x							
	3		x	x	18			-	one	-	-
	4		x	x							
	5		x	x							
	6		x	x	19			-	-	-	-
	7		x	x	20			-	two	-	two/one*
	8		x	x							
	9		x								
	10		x		21			one	two	-	two
2	11		x	x							
	12	x	x		22			one	one	one	-
	13	x	x		23			-	-		
	14		x		24	-	two	-	-		
	15		x		25	-	two	-	-		
	16-1		x		26			-	-		
	16-2		x		27			-	one		
	17	x	x		28			-	-		
	18		x		29	-	two	-	-		
	19		x	x	30			-	-		
	20		x		31			-	-	-	-
	21		x		32			-	-		
	22-1		x		33			-	-		
	22-2		x		34			-	one		

Supplementary Table S1 (Continued)

Sessions	Days	Daily composition			Faecal samples	DNA metabarcoding detections					
						Prey items		Pet food		Surplus meat/fish	
		Prey items	Pet food	Surplus meat/fish		Ion PGM	MiSeq	Ion PGM	MiSeq	Ion PGM	MiSeq
	23-1		x		35			-	-		
	23-2		x		36			-	-		
	24		x		37			one	-		
2	25-1		x		38			-	-		
	25-2		x		39			-	-		
	26		x		40			-	two		
	27		x		41			-	-		

* The one replicate occurrence was associated to surplus raw pig meat while the two replicates occurrence was associated to the surplus raw sheep meat.

Supplementary Table S2: Detailed outputs from Ion PGM and MiSeq platforms during the field study.

Similarity thresholds used to identify variants and observed occurrences of food items in 326 field-collected faecal samples from free-ranging cats. Similarity thresholds and observed occurrences are shown according to the food item and the HTS platform.

Taxa			Ion PGM run						MiSeq run					
			Similarity thresholds			Observed occurrence			Similarity thresholds			Observed occurrence		
**Food category**	**Food sub-category**	**Food items**	95%	98%	100%	in one replicate	in two replicates	Total	95%	98%	100%	in one replicate	in two replicates	Total
**Preys items**	**small / medium-sized rodents**	*Apodemus flavicollis*			x	2	2	4			x	2	2	4
		Apodemus sylvaticus			x	0	4	4			x	3	2	5
		Arvicola terrestris			x	28	47	75			x	23	56	79
		Micromys minutus			x	0	2	2			x	1	2	3
		Microtus arvalis			x	38	56	94			x	60	29	89
		Microtus agrestis			x	7	13	20			x	15	12	27
		Microtus spp.			x	5	3	8			x	1	1	2
		Mus musculus			x	1	0	1			x	1	0	1
		Myodes glareolus			x	7	6	13			x	8	2	10
		Rattus norvegicus	-	-	-	0	0	0			x	3	2	5
		Rattus spp.	-	-	-	0	0	0			x	2	3	5
	**insectivores**	*Crocidura leucodon*			x	1	0	1	-	-	-	0	0	0
		Crocidura russula			x	0	2	2			x	6	2	8
		Sorex coronatus			x	0	1	1			x	1	0	1
	**wild birds**	*Carduelis carduelis*	-	-	-	0	0	0			x	1	1	2
		Columba livia			x	1	0	1			x	0	1	1
		Columba palumbus			x	0	1	1			x	0	1	1
		Delichon urbicum	-	-	-	0	0	0			x	1	0	1
		Falco sp.			x	0	1	1			x	1	1	2
		Hirondo rustica			x	1	0	1			x	0	2	2
		Passer domesticus	-	-	-	0	0	0		x		1	0	1

Supplementary Table S2 (Continued)

Taxa			Ion PGM run						MiSeq run					
**Food category**	**Food sub-category**	**Food items**	**Similarity thresholds**			**Observed occurrence**			**Similarity thresholds**			**Observed occurrence**		
			95%	98%	100%	in one replicate	in two replicates	Total	95%	98%	100%	in one replicate	in two replicates	Total
**Preys items**	**wild birds**	*Passer montanus*	-	-	-	0	0	0			x	2	0	2
		Passer spp.	-	-	-	0	0	0			x	10	7	17
		Pica pica	-	-	-	0	0	0			x	1	0	1
		Streptopelia decaocto	-	-	-	0	0	0			x	3	0	3
		Sylvia atricapilla	-	-	-	0	0	0		x		1	0	1
		Turdus philomelos			x	0	1	1	-	-	-	0	0	0
		Turdus pilaris			x	1	0	1			x	2	0	2
	**reptiles**	*Anguis fragilis*	-	-	-	0	0	0			x	1	0	1
**Human - linked food**	**game species**	*Capreolus capreolus*			x	1	1	2		x		1	1	2
		Cervus elaphus	-	-	-	0	0	0		x		1	0	1
		Perdicinae*	-	-	-	0	0	0			x	3	3	6
		*Oryctolagus cuniculus**			x	3	0	3			x	3	2	5
	**anthropogenic food**	Acanthomorpha	x			4	0	4		x		2	1	3
		Anser sp.	-	-	-	0	0	0		x		3	0	3
		Anseriformes	-	-	-	0	0	0		x		9	3	12
		Bos frontalis		x		1	0	1	-	-	-	0	0	0
		Bos indicus		x		1	2	3		x		4	0	4
		Bos sp.		x		60	34	94		x		46	40	86
		Bos taurus		x		3	1	4		x		4	1	5
		Cairina moschata		x		17	7	24		x		14	4	18
		Capra sp.		x		0	1	1	x			1	0	1
		Caprinae	x			1	0	1	x			18	5	23
		Clupea harengus		x		1	0	1	-	-	-	0	0	0

Supplementary Table S2 (Continued)

Taxa			Ion PGM run						MiSeq run					
Food category	Food sub-category	Food items	Similarity thresholds			Observed occurrence			Similarity thresholds			Observed occurrence		
			95%	98%	100%	in one replicate	in two replicates	Total	95%	98%	100%	in one replicate	in two replicates	Total
		Clupea sp.	-	-	-	0	0	0		x		1	0	1
		Equus sp.	x			1	0	1	-	-	-	0	0	0
		Esox lucius		x		1	0	1	-	-	-	0	0	0
		Gallus gallus domesticus	-	-	-	0	0	0	x			1	0	1
		Gallus sp.	-	-	-	0	0	0		x		43	34	77
		Katsuwonus pelamis		x		2	0	2	-	-	-	0	0	0
		Meleagris gallopavo		x		4	1	5		x		25	14	39
**Human - linked food**	**anthropogenic food**	*Merlangius merlangus*		x		1	0	1	-	-	-	0	0	0
		Neognathae - Galloanserae	-	-	-	0	0	0	x			33	21	54
		Numida meleagris	-	-	-	0	0	0		x		8	2	10
		Ovis aries		x		2	2	4	x			0	1	1
		Ovis sp.		x		8	0	8		x		18	4	22
		Percomorpha	-	-	-	0	0	0	x			1	0	1
		Salmo salar		x		2	2	4		x		3	1	4
		Suidae	-	-	-	0	0	0		x		8	0	8
		Sus scrofa domesticus		x		8	3	11		x		31	19	50
		Sus sp.	-	-	-	0	0	0		x		15	1	16
		Thunnus sp.	-	-	-	0	0	0		x		1	0	1

* Only available from pet food or human leftovers (rabbit hutches and hunting scraps) for cats in this area.

Supplementary Table S3: Variables used in analyses.

Variables used in the evaluation of HTS platforms efficiency for the food item detection.

**Explanatory variables**	**Type**	**Description**
fPlatform	Categorical	HTS platforms used: (1) Ion PGM; (2) Illumina MiSeq
fFoodItem	Categorical	Food item categories: (1) Human-linked food (HLF); (2) Water vole (WV) ; (3) Field vole (FV) ; (4) Common vole (CV) ; (5) Microtus-like vole (MLV); (6) Murids (harvest mouse, yellow-necked mouse, forest mouse, house mouse, rats *Rattus spp.* and Norway rats); (7) Bank vole (BV); (8) Birds (Falconiformes, Columbiformes and Passeriformes, BIR); (9) Shrews (Soricidae *spp.*, SH)
fSample	Categorical	Number code identifying field-collected faecal samples individually
**Response variables**	**Type**	**Description**
Occurrence	Binary	Food item successfully (1) or unsuccessfully (2) detected

Supplementary Table S4: Modelling outputs.

Models explaining the probability of detecting a food item in field-collected faeces of free-ranging cats.

Rank	Model description	K	LL	AIC	∆AIC
1	fPlatform+ fFoodItem+ fPlatform : fFoodItem	18	-435.0	906.8	0.00
2	fPlatform + fFoodItem	10	-473.6	967.4	60.6
3	fFoodItem	9	-507.1	1032.4	125.6
4	Null	1	-526.0	1054.0	147.2

Supplementary Table S5: List of prey items and surplus products given to the cat during the calibration study

Sessions	Days	Daily composition		
		Prey items	Pet food	Surplus meat/fish
1	1		x	
	2		x	
	3	1 *Arvicola terrestris*	x	
	4	1 *Arvicola terrestris*<br>1 *Apodemus sp.*	x	
	5	1 *Arvicola terrestris*	x	
	6	3 *Microtus agrestis*	x	
	7	1 *Apodemus sp.*<br>1 *Mus musculus*	x	
	8	1 *Rattus rattus*		
	9		x	
	10		x	
	11		x	
	12		x	
	13		x	
	14		x	
	15-1		x	
	15-2		x	
	16		x	
	17		x	
	18		x	
	19		x	
	20		x	
	21		x	
	22		x	
2	1		x	
	2		x	herring
	3		x	chicken
	4		x	cod<br>lamb
	5		x	dogfish<br>pork
	6		x	plaice<br>veal
	7		x	duck<br>beef
	8		x	pork<br>beef

Supplementary Table S5 (continued)

Sessions	Days	Daily composition		
		Prey items	Pet food	Surplus meat/fish
	9		x	
	10		x	
	11		x	duck
	12	1 *Taeniopygia guttata*	x	
	13	1 *Taeniopygia guttata*	x	
	14		x	
	15		x	
	16-1		x	
	16-2		x	
	17	1 *Mus musculus*	x	
	18		x	
2	19		x	wild boar
	20		x	
	21		x	
	22-1		x	
	22-2		x	
	23-1		x	
	23-2			
	24		x	
	25-1		x	
	25-2		x	
	26		x	
	27		x	
