# Supplementary material for: Evaluating metabarcoding to analyse diet composition of species foraging in anthropogenic landscapes using Ion Torrent and Illumina sequencing
Source: Sci Rep. 2018 Nov 20;8:17091. doi: 10.1038/s41598-018-34430-7 (PMC6244006; doi:10.1038/s41598-018-34430-7)
Supplement: Supplementary file 2 — Detailed list of aligned sequences [file 41598_2018_34430_MOESM2_ESM.pdf]

Evaluating metabarcoding to analyse diet composition of species foraging in anthropogenic landscapes using Ion Torrent and Illumina sequencing

M.-A. Forin-Wiart, M.-L. Poulle, S. Piry, F. Cosson, C. Larose and M. Galan.

GenBank Accession N°	Genus	species	subspecies
AF159391.1	Apodemus	alpicola	
AB032854.1	Apodemus	alpicola	
AJ311152.1	Apodemus	alpicola	
AJ311153.1	Apodemus	alpicola	
AF159392.1	Apodemus	flavicollis	
AY158443.1	Apodemus	flavicollis	
AY158444.1	Apodemus	flavicollis	
AY158445.1	Apodemus	flavicollis	
AY158450.1	Apodemus	flavicollis	
AY158451.1	Apodemus	flavicollis	
AY158452.1	Apodemus	flavicollis	
AY158453.1	Apodemus	flavicollis	
AB032853.1	Apodemus	flavicollis	
AJ311150.1	Apodemus	flavicollis	
AJ311151.1	Apodemus	flavicollis	
AJ605644.1	Apodemus	flavicollis	
AJ605642.1	Apodemus	flavicollis	
AJ605643.1	Apodemus	flavicollis	
AJ605657.1	Apodemus	flavicollis	
AJ605661.1	Apodemus	flavicollis	
AJ605660.1	Apodemus	flavicollis	
AJ605691.1	Apodemus	flavicollis	
AJ605640.1	Apodemus	flavicollis	
AJ605600.1	Apodemus	flavicollis	
AJ605616.1	Apodemus	flavicollis	
AJ605629.1	Apodemus	flavicollis	
AJ605631.1	Apodemus	flavicollis	
AJ605633.1	Apodemus	flavicollis	
AJ605672.1	Apodemus	flavicollis	
AJ605667.1	Apodemus	flavicollis	
AJ605668.1	Apodemus	flavicollis	
AJ605676.1	Apodemus	flavicollis	
AJ605677.1	Apodemus	flavicollis	
AJ605688.1	Apodemus	flavicollis	
AJ605690.1	Apodemus	flavicollis	
AJ605605.1	Apodemus	flavicollis	
AJ605666.1	Apodemus	flavicollis	
AJ605652.1	Apodemus	flavicollis	
AJ605664.1	Apodemus	flavicollis	
AJ605663.1	Apodemus	flavicollis	
AJ605634.1	Apodemus	flavicollis	
AJ605601.1	Apodemus	flavicollis	
AJ605604.1	Apodemus	flavicollis	

AJ605665.1	Apodemus	flavicollis	
AJ605641.1	Apodemus	flavicollis	
AJ605610.1	Apodemus	flavicollis	
AJ605609.1	Apodemus	flavicollis	
AJ605606.1	Apodemus	flavicollis	
AJ605608.1	Apodemus	flavicollis	
AJ605614.1	Apodemus	flavicollis	
AJ605651.1	Apodemus	flavicollis	
AJ605650.1	Apodemus	flavicollis	
AJ605656.1	Apodemus	flavicollis	
AJ605625.1	Apodemus	flavicollis	
AJ605624.1	Apodemus	flavicollis	
AJ605646.1	Apodemus	flavicollis	
AJ605647.1	Apodemus	flavicollis	
AJ605622.1	Apodemus	flavicollis	
AJ605621.1	Apodemus	flavicollis	
AJ605620.1	Apodemus	flavicollis	
AJ605619.1	Apodemus	flavicollis	
AJ605618.1	Apodemus	flavicollis	
AJ605617.1	Apodemus	flavicollis	
AJ605686.1	Apodemus	flavicollis	
AJ298604.1	Apodemus	flavicollis	
AJ298603.1	Apodemus	flavicollis	
AJ298601.1	Apodemus	flavicollis	
AJ298602.1	Apodemus	flavicollis	
AF159395.1	Apodemus	sylvaticus	
AF429819.1	Apodemus	sylvaticus	
AF429820.1	Apodemus	sylvaticus	
HQ158102.1	Apodemus	sylvaticus	
AY158456.1	Apodemus	sylvaticus	
AY158461.1	Apodemus	sylvaticus	
AB033695.1	Apodemus	sylvaticus	
AJ311149.1	Apodemus	sylvaticus	
AJ311148.1	Apodemus	sylvaticus	
AJ511903.1	Apodemus	sylvaticus	
AJ511881.1	Apodemus	sylvaticus	
AJ511902.1	Apodemus	sylvaticus	
AJ511923.1	Apodemus	sylvaticus	
AJ511942.1	Apodemus	sylvaticus	
AJ511943.1	Apodemus	sylvaticus	
AJ511936.1	Apodemus	sylvaticus	
AJ511938.1	Apodemus	sylvaticus	
AJ511922.1	Apodemus	sylvaticus	
AJ511921.1	Apodemus	sylvaticus	

AJ511916.1	Apodemus	sylvaticus	
AJ511946.1	Apodemus	sylvaticus	
AJ511945.1	Apodemus	sylvaticus	
AJ511905.1	Apodemus	sylvaticus	
AJ511885.1	Apodemus	sylvaticus	
AJ511901.1	Apodemus	sylvaticus	
AJ511908.1	Apodemus	sylvaticus	
AJ511899.1	Apodemus	sylvaticus	
AJ511891.1	Apodemus	sylvaticus	
AJ511911.1	Apodemus	sylvaticus	
AJ511912.1	Apodemus	sylvaticus	
AJ511910.1	Apodemus	sylvaticus	
AJ511948.1	Apodemus	sylvaticus	
AJ511949.1	Apodemus	sylvaticus	
AJ511950.1	Apodemus	sylvaticus	
AJ511962.1	Apodemus	sylvaticus	
AJ511963.1	Apodemus	sylvaticus	
AJ511964.1	Apodemus	sylvaticus	
AJ511965.1	Apodemus	sylvaticus	
AJ511966.1	Apodemus	sylvaticus	
AJ511967.1	Apodemus	sylvaticus	
AJ511951.1	Apodemus	sylvaticus	
AJ511952.1	Apodemus	sylvaticus	
AJ511954.1	Apodemus	sylvaticus	
AJ511955.1	Apodemus	sylvaticus	
AJ511957.1	Apodemus	sylvaticus	
AJ511956.1	Apodemus	sylvaticus	
AJ511968.1	Apodemus	sylvaticus	
AJ511969.1	Apodemus	sylvaticus	
AJ511971.1	Apodemus	sylvaticus	
AJ511958.1	Apodemus	sylvaticus	
AJ511972.1	Apodemus	sylvaticus	
AJ511973.1	Apodemus	sylvaticus	
AJ298598.1	Apodemus	sylvaticus	
AJ298599.1	Apodemus	sylvaticus	
AJ298600.1	Apodemus	sylvaticus	
AY389021.1	Apodemus	uralensis	
AB096837.1	Apodemus	uralensis	
AJ311155.1	Apodemus	uralensis	
AY324476.1	Apomys	camiguinensis	
AY324477.1	Apomys	camiguinensis	
AY324463.1	Apomys	datae	
AY324464.1	Apomys	datae	
AY324465.1	Apomys	gracilostris	

AY324466.1	Apomys	gracilostris	
EU349735.1	Apomys	hylocoetes	
AY324467.1	Apomys	hylocoetes	
AY324468.1	Apomys	hylocoetes	
AY324469.1	Apomys	hylocoetes	
DQ191467.1	Apomys	insignis	
AY324470.1	Apomys	insignis	
AY324471.1	Apomys	insignis	
AY324473.1	Apomys	insignis	
AY324472.1	Apomys	insignis	
DQ191468.1	Apomys	microdon	
AY324478.1	Apomys	microdon	
AY324479.1	Apomys	microdon	
AY324480.1	Apomys	microdon	
AY324481.1	Apomys	microdon	
DQ191469.1	Apomys	musculus	
AY324482.1	Apomys	musculus	
AY324484.1	Apomys	sp.	
AY324485.1	Apomys	sp.	
AY324486.1	Apomys	sp.	
AY324487.1	Apomys	sp.	
AY324488.1	Apomys	sp.	
AY324489.1	Apomys	sp.	
AY324475.1	Apomys	sp.	
AY324474.1	Apomys	sp.	
FJ539346.1	Arvicola	sapidus	
FJ539343.1	Arvicola	sapidus	
FJ539344.1	Arvicola	sapidus	
FJ539345.1	Arvicola	sapidus	
FJ539341.1	Arvicola	sapidus	
FJ539342.1	Arvicola	sapidus	
AF159400.1	Arvicola	terrestris	
AF119269.1	Arvicola	terrestris	
DQ663669.1	Arvicola	terrestris	
AY275106.1	Arvicola	terrestris	
GU954310.1	Arvicola	terrestris	
DQ088704.1	Castor	fiber	
DQ088708.1	Castor	fiber	
DQ088706.1	Castor	fiber	
DQ088705.1	Castor	fiber	
AJ389529.1	Castor	fiber	
DQ088707.1	Castor	fiber	
AY513845.1	Chionomys	nivalis	
AY513846.1	Chionomys	nivalis	

AY513847.1	Chionomys	nivalis	
AY513848.1	Chionomys	nivalis	
AY513849.1	Chionomys	nivalis	
GQ150786.1	Chionomys	nivalis	
GQ150794.1	Chionomys	nivalis	
GQ150795.1	Chionomys	nivalis	
GQ150796.1	Chionomys	nivalis	
GQ150797.1	Chionomys	nivalis	
GQ150798.1	Chionomys	nivalis	
GQ150799.1	Chionomys	nivalis	
GQ150800.1	Chionomys	nivalis	
GQ150787.1	Chionomys	nivalis	
GQ150801.1	Chionomys	nivalis	
GQ150802.1	Chionomys	nivalis	
GQ150788.1	Chionomys	nivalis	
GQ150789.1	Chionomys	nivalis	
GQ150790.1	Chionomys	nivalis	
GQ150791.1	Chionomys	nivalis	
GQ150792.1	Chionomys	nivalis	
GQ150793.1	Chionomys	nivalis	
AM392367.1	Chionomys	nivalis	
AF159401.1	Clethrionomys	glareolus	
AF119272.1	Clethrionomys	glareolus	
DQ663670.1	Clethrionomys	glareolus	
DQ472232.1	Clethrionomys	glareolus	
DQ472233.1	Clethrionomys	glareolus	
DQ472234.1	Clethrionomys	glareolus	
DQ472235.1	Clethrionomys	glareolus	
DQ472236.1	Clethrionomys	glareolus	
DQ472237.1	Clethrionomys	glareolus	
DQ472238.1	Clethrionomys	glareolus	
DQ472239.1	Clethrionomys	glareolus	
DQ472240.1	Clethrionomys	glareolus	
DQ472241.1	Clethrionomys	glareolus	
DQ472346.1	Clethrionomys	glareolus	
DQ472347.1	Clethrionomys	glareolus	
DQ472348.1	Clethrionomys	glareolus	
DQ472230.1	Clethrionomys	glareolus	
DQ472231.1	Clethrionomys	glareolus	
DQ472242.1	Clethrionomys	glareolus	
DQ472243.1	Clethrionomys	glareolus	
DQ472244.1	Clethrionomys	glareolus	
DQ472245.1	Clethrionomys	glareolus	
DQ472246.1	Clethrionomys	glareolus	

DQ472247.1	Clethrionomys	glareolus	
DQ472248.1	Clethrionomys	glareolus	
DQ472249.1	Clethrionomys	glareolus	
DQ472250.1	Clethrionomys	glareolus	
DQ472251.1	Clethrionomys	glareolus	
DQ472252.1	Clethrionomys	glareolus	
DQ472253.1	Clethrionomys	glareolus	
DQ472254.1	Clethrionomys	glareolus	
DQ472255.1	Clethrionomys	glareolus	
DQ472256.1	Clethrionomys	glareolus	
DQ472257.1	Clethrionomys	glareolus	
DQ472258.1	Clethrionomys	glareolus	
DQ472259.1	Clethrionomys	glareolus	
DQ472260.1	Clethrionomys	glareolus	
DQ472261.1	Clethrionomys	glareolus	
DQ472262.1	Clethrionomys	glareolus	
DQ472263.1	Clethrionomys	glareolus	
DQ472264.1	Clethrionomys	glareolus	
DQ472265.1	Clethrionomys	glareolus	
DQ472266.1	Clethrionomys	glareolus	
DQ472267.1	Clethrionomys	glareolus	
DQ472268.1	Clethrionomys	glareolus	
DQ472269.1	Clethrionomys	glareolus	
DQ472270.1	Clethrionomys	glareolus	
DQ472271.1	Clethrionomys	glareolus	
DQ472272.1	Clethrionomys	glareolus	
DQ472273.1	Clethrionomys	glareolus	
DQ472274.1	Clethrionomys	glareolus	
DQ472275.1	Clethrionomys	glareolus	
DQ472276.1	Clethrionomys	glareolus	
DQ472277.1	Clethrionomys	glareolus	
DQ472278.1	Clethrionomys	glareolus	
DQ472279.1	Clethrionomys	glareolus	
DQ472280.1	Clethrionomys	glareolus	
DQ472281.1	Clethrionomys	glareolus	
DQ472282.1	Clethrionomys	glareolus	
DQ472283.1	Clethrionomys	glareolus	
DQ472284.1	Clethrionomys	glareolus	
DQ472285.1	Clethrionomys	glareolus	
DQ472286.1	Clethrionomys	glareolus	
DQ472287.1	Clethrionomys	glareolus	
DQ472288.1	Clethrionomys	glareolus	
DQ472289.1	Clethrionomys	glareolus	
DQ472290.1	Clethrionomys	glareolus	

DQ472291.1	Clethrionomys	glareolus	
DQ472292.1	Clethrionomys	glareolus	
DQ472293.1	Clethrionomys	glareolus	
DQ472294.1	Clethrionomys	glareolus	
DQ472295.1	Clethrionomys	glareolus	
DQ472296.1	Clethrionomys	glareolus	
DQ472297.1	Clethrionomys	glareolus	
DQ472298.1	Clethrionomys	glareolus	
DQ472299.1	Clethrionomys	glareolus	
DQ472300.1	Clethrionomys	glareolus	
DQ472301.1	Clethrionomys	glareolus	
DQ472302.1	Clethrionomys	glareolus	
DQ472303.1	Clethrionomys	glareolus	
DQ472304.1	Clethrionomys	glareolus	
DQ472305.1	Clethrionomys	glareolus	
DQ472306.1	Clethrionomys	glareolus	
DQ472307.1	Clethrionomys	glareolus	
DQ472308.1	Clethrionomys	glareolus	
DQ472309.1	Clethrionomys	glareolus	
DQ472310.1	Clethrionomys	glareolus	
DQ472311.1	Clethrionomys	glareolus	
DQ472312.1	Clethrionomys	glareolus	
DQ472313.1	Clethrionomys	glareolus	
DQ472314.1	Clethrionomys	glareolus	
DQ472315.1	Clethrionomys	glareolus	
DQ472316.1	Clethrionomys	glareolus	
DQ472317.1	Clethrionomys	glareolus	
DQ472318.1	Clethrionomys	glareolus	
DQ472319.1	Clethrionomys	glareolus	
DQ472320.1	Clethrionomys	glareolus	
DQ472321.1	Clethrionomys	glareolus	
DQ472322.1	Clethrionomys	glareolus	
DQ472323.1	Clethrionomys	glareolus	
DQ472324.1	Clethrionomys	glareolus	
DQ472325.1	Clethrionomys	glareolus	
DQ472326.1	Clethrionomys	glareolus	
DQ472327.1	Clethrionomys	glareolus	
DQ472328.1	Clethrionomys	glareolus	
DQ472329.1	Clethrionomys	glareolus	
DQ472330.1	Clethrionomys	glareolus	
DQ472331.1	Clethrionomys	glareolus	
DQ472332.1	Clethrionomys	glareolus	
DQ472333.1	Clethrionomys	glareolus	
DQ472334.1	Clethrionomys	glareolus	

DQ472335.1	Clethrionomys	glareolus	
DQ472336.1	Clethrionomys	glareolus	
DQ472337.1	Clethrionomys	glareolus	
DQ472338.1	Clethrionomys	glareolus	
DQ472339.1	Clethrionomys	glareolus	
DQ472340.1	Clethrionomys	glareolus	
DQ472341.1	Clethrionomys	glareolus	
DQ472342.1	Clethrionomys	glareolus	
DQ472343.1	Clethrionomys	glareolus	
DQ472344.1	Clethrionomys	glareolus	
DQ472345.1	Clethrionomys	glareolus	
AF429812.1	Clethrionomys	glareolus	
AF429813.1	Clethrionomys	glareolus	
AY185800.1	Clethrionomys	glareolus	
AY185796.1	Clethrionomys	glareolus	
AY185798.1	Clethrionomys	glareolus	
AY062901.1	Clethrionomys	glareolus	
AY062902.1	Clethrionomys	glareolus	
AF367075.1	Clethrionomys	glareolus	
AY062903.1	Clethrionomys	glareolus	
AY062904.1	Clethrionomys	glareolus	
AF367080.1	Clethrionomys	glareolus	
AF367079.1	Clethrionomys	glareolus	
AF429810.1	Clethrionomys	glareolus	
AY062905.1	Clethrionomys	glareolus	
AF367076.1	Clethrionomys	glareolus	
AY062906.1	Clethrionomys	glareolus	
AF429781.1	Clethrionomys	glareolus	
AF429811.1	Clethrionomys	glareolus	
AY062907.1	Clethrionomys	glareolus	
AF429782.1	Clethrionomys	glareolus	
AF429798.1	Clethrionomys	glareolus	
AF429783.1	Clethrionomys	glareolus	
AF429784.1	Clethrionomys	glareolus	
AF429785.1	Clethrionomys	glareolus	
AF429786.1	Clethrionomys	glareolus	
AF429787.1	Clethrionomys	glareolus	
AF429788.1	Clethrionomys	glareolus	
AF367074.1	Clethrionomys	glareolus	
AF367081.1	Clethrionomys	glareolus	
AF429799.1	Clethrionomys	glareolus	
AF429800.1	Clethrionomys	glareolus	
AF367082.1	Clethrionomys	glareolus	
AF429795.1	Clethrionomys	glareolus	

AF429796.1	Clethrionomys	glareolus	
AF429789.1	Clethrionomys	glareolus	
AF429801.1	Clethrionomys	glareolus	
AF429802.1	Clethrionomys	glareolus	
AF429803.1	Clethrionomys	glareolus	
AF367083.1	Clethrionomys	glareolus	
AF429804.1	Clethrionomys	glareolus	
AF429794.1	Clethrionomys	glareolus	
AF429797.1	Clethrionomys	glareolus	
AF429805.1	Clethrionomys	glareolus	
AF429806.1	Clethrionomys	glareolus	
AF429807.1	Clethrionomys	glareolus	
AF429808.1	Clethrionomys	glareolus	
AF429790.1	Clethrionomys	glareolus	
AF367084.1	Clethrionomys	glareolus	
AF429809.1	Clethrionomys	glareolus	
AY062900.1	Clethrionomys	glareolus	
AM392368.1	Clethrionomys	glareolus	
AJ639699.1	Clethrionomys	glareolus	
AJ639700.1	Clethrionomys	glareolus	
AJ639701.1	Clethrionomys	glareolus	
AJ639702.1	Clethrionomys	glareolus	
AJ639703.1	Clethrionomys	glareolus	
AJ639707.1	Clethrionomys	glareolus	
AJ639704.1	Clethrionomys	glareolus	
AJ639705.1	Clethrionomys	glareolus	
AJ639706.1	Clethrionomys	glareolus	
AJ639661.1	Clethrionomys	glareolus	
AJ639686.2	Clethrionomys	glareolus	
AJ639688.1	Clethrionomys	glareolus	
AJ639689.1	Clethrionomys	glareolus	
AJ639685.1	Clethrionomys	glareolus	
AJ639683.2	Clethrionomys	glareolus	
AJ639680.2	Clethrionomys	glareolus	
AJ639682.1	Clethrionomys	glareolus	
AJ639679.1	Clethrionomys	glareolus	
AJ639678.1	Clethrionomys	glareolus	
AJ639691.2	Clethrionomys	glareolus	
AJ639681.2	Clethrionomys	glareolus	
AJ639692.1	Clethrionomys	glareolus	
AJ639677.1	Clethrionomys	glareolus	
AJ639693.1	Clethrionomys	glareolus	
AJ639675.1	Clethrionomys	glareolus	
AJ639674.1	Clethrionomys	glareolus	

AJ639673.2	Clethrionomys	glareolus	
AJ639672.1	Clethrionomys	glareolus	
AJ639695.1	Clethrionomys	glareolus	
AJ639684.1	Clethrionomys	glareolus	
AJ639696.1	Clethrionomys	glareolus	
AJ639667.1	Clethrionomys	glareolus	
AJ639665.1	Clethrionomys	glareolus	
AJ639664.1	Clethrionomys	glareolus	
AJ639663.1	Clethrionomys	glareolus	
AJ639662.1	Clethrionomys	glareolus	
AJ639708.1	Clethrionomys	glareolus	
AJ867965.1	Clethrionomys	glareolus	
AJ867966.1	Clethrionomys	glareolus	
AJ867967.1	Clethrionomys	glareolus	
AJ867969.1	Clethrionomys	glareolus	
AJ867970.1	Clethrionomys	glareolus	
AJ867971.1	Clethrionomys	glareolus	
AJ867972.1	Clethrionomys	glareolus	
AJ867979.1	Clethrionomys	glareolus	
AJ867948.1	Clethrionomys	glareolus	
AJ867959.1	Clethrionomys	glareolus	
AJ867980.1	Clethrionomys	glareolus	
AJ867973.1	Clethrionomys	glareolus	
AJ867974.1	Clethrionomys	glareolus	
AJ867976.1	Clethrionomys	glareolus	
AJ867977.1	Clethrionomys	glareolus	
AJ867975.1	Clethrionomys	glareolus	
AJ867950.1	Clethrionomys	glareolus	
AJ867956.1	Clethrionomys	glareolus	
AJ867960.1	Clethrionomys	glareolus	
AJ867951.1	Clethrionomys	glareolus	
AJ867961.1	Clethrionomys	glareolus	
AJ867981.1	Clethrionomys	glareolus	
AJ867962.1	Clethrionomys	glareolus	
AJ867957.1	Clethrionomys	glareolus	
AJ867952.1	Clethrionomys	glareolus	
AJ867958.1	Clethrionomys	glareolus	
AJ867963.1	Clethrionomys	glareolus	
AJ867949.1	Clethrionomys	glareolus	
AJ867953.1	Clethrionomys	glareolus	
AJ867954.1	Clethrionomys	glareolus	
AJ867955.1	Clethrionomys	glareolus	
AJ867978.1	Clethrionomys	glareolus	
DQ389591.1	Clethrionomys	glareolus	

DQ389580.1	Clethrionomys	glareolus	
DQ389581.1	Clethrionomys	glareolus	
AF318584.1	Clethrionomys	glareolus	
AF318585.1	Clethrionomys	glareolus	
AY309420.1	Clethrionomys	glareolus	
AY309421.1	Clethrionomys	glareolus	
DQ408266.1	Clethrionomys	glareolus	
DQ408267.1	Clethrionomys	glareolus	
DQ408277.1	Clethrionomys	glareolus	
DQ408279.1	Clethrionomys	glareolus	
DQ408280.1	Clethrionomys	glareolus	
DQ408281.1	Clethrionomys	glareolus	
DQ408282.1	Clethrionomys	glareolus	
DQ408283.1	Clethrionomys	glareolus	
DQ408284.1	Clethrionomys	glareolus	
DQ408285.1	Clethrionomys	glareolus	
DQ408286.1	Clethrionomys	glareolus	
DQ408287.1	Clethrionomys	glareolus	
DQ408288.1	Clethrionomys	glareolus	
DQ408289.1	Clethrionomys	glareolus	
DQ408290.1	Clethrionomys	glareolus	
DQ408291.1	Clethrionomys	glareolus	
DQ408292.1	Clethrionomys	glareolus	
DQ408293.1	Clethrionomys	glareolus	
DQ408278.1	Clethrionomys	glareolus	
DQ408265.1	Clethrionomys	glareolus	
DQ408264.1	Clethrionomys	glareolus	
DQ393795.1	Clethrionomys	glareolus	
DQ393796.1	Clethrionomys	glareolus	
DQ393797.1	Clethrionomys	glareolus	
DQ393798.1	Clethrionomys	glareolus	
DQ393799.1	Clethrionomys	glareolus	
DQ393800.1	Clethrionomys	glareolus	
DQ393801.1	Clethrionomys	glareolus	
DQ393802.1	Clethrionomys	glareolus	
DQ393803.1	Clethrionomys	glareolus	
DQ393804.1	Clethrionomys	glareolus	
DQ393805.1	Clethrionomys	glareolus	
AY275109.1	Cricetus	cricetus	
EU107523.1	Cricetus	cricetus	
EU107532.1	Cricetus	cricetus	
EU107533.1	Cricetus	cricetus	
EU107534.1	Cricetus	cricetus	
EU107535.1	Cricetus	cricetus	

EU107524.1	Cricetus	cricetus	
EU107525.1	Cricetus	cricetus	
EU107526.1	Cricetus	cricetus	
EU107527.1	Cricetus	cricetus	
EU107528.1	Cricetus	cricetus	
EU107529.1	Cricetus	cricetus	
EU107530.1	Cricetus	cricetus	
EU107531.1	Cricetus	cricetus	
AJ973392.1	Cricetus	cricetus	
AJ633756.1	Cricetus	cricetus	
AJ633757.1	Cricetus	cricetus	
AJ633758.1	Cricetus	cricetus	
AJ633759.1	Cricetus	cricetus	
AJ633760.1	Cricetus	cricetus	
AJ633761.1	Cricetus	cricetus	
AJ633762.1	Cricetus	cricetus	
AJ633763.1	Cricetus	cricetus	
AJ633764.1	Cricetus	cricetus	
AJ633765.1	Cricetus	cricetus	
AJ633766.1	Cricetus	cricetus	
AJ633767.1	Cricetus	cricetus	
AJ633768.1	Cricetus	cricetus	
AJ633769.1	Cricetus	cricetus	
AJ633770.1	Cricetus	cricetus	
AJ633771.1	Cricetus	cricetus	
AJ633772.1	Cricetus	cricetus	
AJ633773.1	Cricetus	cricetus	
AJ633774.1	Cricetus	cricetus	
AJ633775.1	Cricetus	cricetus	
AJ633776.1	Cricetus	cricetus	
AJ633777.1	Cricetus	cricetus	
AJ633779.1	Cricetus	cricetus	
AJ633780.1	Cricetus	cricetus	
AJ633782.1	Cricetus	cricetus	
AJ490302.1	Cricetus	cricetus	
AJ490303.1	Cricetus	cricetus	
AJ490304.1	Cricetus	cricetus	
AJ490305.1	Cricetus	cricetus	
AJ490306.1	Cricetus	cricetus	
AJ490307.1	Cricetus	cricetus	
AJ490308.1	Cricetus	cricetus	
AJ490309.1	Cricetus	cricetus	
AJ490310.1	Cricetus	cricetus	
AJ225030.1	Eliomys	quercinus	

GQ453668.1	Eliomys	quercinus	
GQ453669.1	Eliomys	quercinus	
AJ225031.1	Glis	glis	
AF143929.1	Marmota	marmota	
AF143930.1	Marmota	marmota	
AF100711.1	Marmota	marmota	
AF159399.1	Micromys	minutus	
AB033697.1	Micromys	minutus	
AB201984.1	Micromys	minutus	
AB201985.1	Micromys	minutus	
AB201976.1	Micromys	minutus	
AB201981.1	Micromys	minutus	
AB201982.1	Micromys	minutus	
AB201983.1	Micromys	minutus	
AB201967.1	Micromys	minutus	
AB201966.1	Micromys	minutus	
AB201968.1	Micromys	minutus	
AB201965.1	Micromys	minutus	
AB201959.1	Micromys	minutus	
AB201986.1	Micromys	minutus	
AB201987.1	Micromys	minutus	
AB201989.1	Micromys	minutus	
AB201990.1	Micromys	minutus	
AB201991.1	Micromys	minutus	
AB201992.1	Micromys	minutus	
AB201993.1	Micromys	minutus	
AB201994.1	Micromys	minutus	
AB201995.1	Micromys	minutus	
AB201988.1	Micromys	minutus	
AB201996.1	Micromys	minutus	
AB201975.1	Micromys	minutus	
AB201969.1	Micromys	minutus	
AB201970.1	Micromys	minutus	
AB201971.1	Micromys	minutus	
AB201972.1	Micromys	minutus	
AB201973.1	Micromys	minutus	
AB201974.1	Micromys	minutus	
AB201979.1	Micromys	minutus	
AB201980.1	Micromys	minutus	
AB201960.1	Micromys	minutus	
AB201961.1	Micromys	minutus	
AB201962.1	Micromys	minutus	
AB201963.1	Micromys	minutus	
AB201958.1	Micromys	minutus	

AB201964.1	Micromys	minutus	
AB201977.1	Micromys	minutus	
AB201978.1	Micromys	minutus	
AB125080.1	Micromys	minutus	
AB125091.1	Micromys	minutus	
AB125092.1	Micromys	minutus	
AB125078.1	Micromys	minutus	
AB125079.1	Micromys	minutus	
AB125093.1	Micromys	minutus	
AB125095.1	Micromys	minutus	
AB125096.1	Micromys	minutus	
AB125097.1	Micromys	minutus	
AB125088.1	Micromys	minutus	
AB125089.1	Micromys	minutus	
AB125090.1	Micromys	minutus	
AB125082.1	Micromys	minutus	
AB125083.1	Micromys	minutus	
AB125085.1	Micromys	minutus	
AB125086.1	Micromys	minutus	
AB125087.1	Micromys	minutus	
AB125069.1	Micromys	minutus	
AB125084.1	Micromys	minutus	
AB125098.1	Micromys	minutus	
AB125099.1	Micromys	minutus	
AB125100.1	Micromys	minutus	
AB125081.1	Micromys	minutus	
AB125070.1	Micromys	minutus	
AB125071.1	Micromys	minutus	
AB125072.1	Micromys	minutus	
AB125073.1	Micromys	minutus	
AB125074.1	Micromys	minutus	
AB125075.1	Micromys	minutus	
AB125076.1	Micromys	minutus	
AB125077.1	Micromys	minutus	
AB125094.1	Micromys	minutus	
HM217360.1	Micromys	minutus	
HM217361.1	Micromys	minutus	
AF119271.1	Microtus	agrestis	
AF159402.1	Microtus	agrestis	
DQ663658.1	Microtus	agrestis	
AY167155.1	Microtus	agrestis	
AY167213.1	Microtus	agrestis	
AY167180.1	Microtus	agrestis	
AY167149.1	Microtus	agrestis	

AY167156.1	Microtus	agrestis	
AY167157.1	Microtus	agrestis	
AY167171.1	Microtus	agrestis	
AY167175.1	Microtus	agrestis	
AY167151.1	Microtus	agrestis	
AY167152.1	Microtus	agrestis	
AY167212.1	Microtus	agrestis	
AY167179.1	Microtus	agrestis	
AY167184.1	Microtus	agrestis	
AY167194.1	Microtus	agrestis	
AY167170.1	Microtus	agrestis	
AY167193.1	Microtus	agrestis	
AY167197.1	Microtus	agrestis	
AY167192.1	Microtus	agrestis	
AY167191.1	Microtus	agrestis	
AY167150.1	Microtus	agrestis	
AY167198.1	Microtus	agrestis	
AY167199.1	Microtus	agrestis	
AY167205.1	Microtus	agrestis	
AY167195.1	Microtus	agrestis	
AY167196.1	Microtus	agrestis	
AY167169.1	Microtus	agrestis	
AY167173.1	Microtus	agrestis	
AY167188.1	Microtus	agrestis	
AY167189.1	Microtus	agrestis	
AY167210.1	Microtus	agrestis	
AY167176.1	Microtus	agrestis	
AY167177.1	Microtus	agrestis	
AY167178.1	Microtus	agrestis	
AY167183.1	Microtus	agrestis	
AY167202.1	Microtus	agrestis	
AY167185.1	Microtus	agrestis	
AY167186.1	Microtus	agrestis	
AY167172.1	Microtus	agrestis	
AY167190.1	Microtus	agrestis	
AY167182.1	Microtus	agrestis	
AY167181.1	Microtus	agrestis	
AY167162.1	Microtus	agrestis	
AY167163.1	Microtus	agrestis	
AY167187.1	Microtus	agrestis	
AY167164.1	Microtus	agrestis	
AY167211.1	Microtus	agrestis	
AY167201.1	Microtus	agrestis	
AY167200.1	Microtus	agrestis	

AY167203.1	Microtus	agrestis	
AY167208.1	Microtus	agrestis	
AY167209.1	Microtus	agrestis	
AY167207.1	Microtus	agrestis	
AY167206.1	Microtus	agrestis	
AY167204.1	Microtus	agrestis	
AY167167.1	Microtus	agrestis	
AY167168.1	Microtus	agrestis	
AY167160.1	Microtus	agrestis	
AY167161.1	Microtus	agrestis	
AY167158.1	Microtus	agrestis	
AY167159.1	Microtus	agrestis	
AY167153.1	Microtus	agrestis	
AY167154.1	Microtus	agrestis	
AY167174.1	Microtus	agrestis	
AY167166.1	Microtus	agrestis	
AY167165.1	Microtus	agrestis	
FJ619775.1	Microtus	agrestis	
FJ619779.1	Microtus	agrestis	
FJ619764.1	Microtus	agrestis	
FJ619765.1	Microtus	agrestis	
FJ619776.1	Microtus	agrestis	
FJ619784.1	Microtus	agrestis	
FJ619777.1	Microtus	agrestis	
FJ619746.1	Microtus	agrestis	
FJ619785.1	Microtus	agrestis	
FJ619778.1	Microtus	agrestis	
FJ619786.1	Microtus	agrestis	
FJ619755.1	Microtus	agrestis	
FJ619756.1	Microtus	agrestis	
FJ619747.1	Microtus	agrestis	
FJ619748.1	Microtus	agrestis	
FJ619749.1	Microtus	agrestis	
FJ619757.1	Microtus	agrestis	
FJ619758.1	Microtus	agrestis	
FJ619766.1	Microtus	agrestis	
FJ619750.1	Microtus	agrestis	
FJ619767.1	Microtus	agrestis	
FJ619751.1	Microtus	agrestis	
FJ619759.1	Microtus	agrestis	
FJ619760.1	Microtus	agrestis	
FJ619768.1	Microtus	agrestis	
FJ619781.1	Microtus	agrestis	
FJ619769.1	Microtus	agrestis	

FJ619752.1	Microtus	agrestis	
FJ619782.1	Microtus	agrestis	
FJ619761.1	Microtus	agrestis	
FJ619770.1	Microtus	agrestis	
FJ619762.1	Microtus	agrestis	
FJ619771.1	Microtus	agrestis	
FJ619753.1	Microtus	agrestis	
FJ619763.1	Microtus	agrestis	
FJ619783.1	Microtus	agrestis	
FJ619772.1	Microtus	agrestis	
FJ619754.1	Microtus	agrestis	
FJ619773.1	Microtus	agrestis	
FJ619774.1	Microtus	agrestis	
FJ619780.1	Microtus	agrestis	
EU439956.1	Microtus	agrestis	
DQ662101.1	Microtus	agrestis	
DQ662102.1	Microtus	agrestis	
DQ768145.1	Microtus	agrestis	
DQ662097.1	Microtus	agrestis	
DQ662098.1	Microtus	agrestis	
DQ662096.1	Microtus	agrestis	
DQ662099.1	Microtus	agrestis	
DQ662095.1	Microtus	agrestis	
DQ662100.1	Microtus	agrestis	
DQ768140.1	Microtus	agrestis	
DQ768141.1	Microtus	agrestis	
DQ480084.1	Microtus	agrestis	
AF159403.1	Microtus	arvalis	
DQ663659.1	Microtus	arvalis	
FJ789987.1	Microtus	arvalis	
AY220761.1	Microtus	arvalis	
AY708460.1	Microtus	arvalis	
AY708461.1	Microtus	arvalis	
GU187363.1	Microtus	arvalis	
GU187372.1	Microtus	arvalis	
GU187373.1	Microtus	arvalis	
GU187374.1	Microtus	arvalis	
GU187375.1	Microtus	arvalis	
GU187376.1	Microtus	arvalis	
GU187377.1	Microtus	arvalis	
GU187378.1	Microtus	arvalis	
GU187379.1	Microtus	arvalis	
GU187380.1	Microtus	arvalis	
GU187364.1	Microtus	arvalis	

GU187365.1	Microtus	arvalis	
GU187366.1	Microtus	arvalis	
GU187367.1	Microtus	arvalis	
GU187369.1	Microtus	arvalis	
GU187368.1	Microtus	arvalis	
GU187370.1	Microtus	arvalis	
GU187371.1	Microtus	arvalis	
AY708508.1	Microtus	arvalis	
AY708509.1	Microtus	arvalis	
AY708510.1	Microtus	arvalis	
AY708462.1	Microtus	arvalis	
FJ789989.1	Microtus	arvalis	
FJ789990.1	Microtus	arvalis	
FJ789991.1	Microtus	arvalis	
FJ789992.1	Microtus	arvalis	
AY708463.1	Microtus	arvalis	
AY708464.1	Microtus	arvalis	
FJ789993.1	Microtus	arvalis	
AY708512.1	Microtus	arvalis	
AY708513.1	Microtus	arvalis	
AY708465.1	Microtus	arvalis	
AY708466.1	Microtus	arvalis	
FJ789994.1	Microtus	arvalis	
FJ789995.1	Microtus	arvalis	
FJ789996.1	Microtus	arvalis	
AY708482.1	Microtus	arvalis	
FJ789997.1	Microtus	arvalis	
FJ789998.1	Microtus	arvalis	
FJ789999.1	Microtus	arvalis	
FJ790000.1	Microtus	arvalis	
FJ790001.1	Microtus	arvalis	
AY708519.1	Microtus	arvalis	
FJ790002.1	Microtus	arvalis	
AY708514.1	Microtus	arvalis	
AY708515.1	Microtus	arvalis	
AY708516.1	Microtus	arvalis	
AY708467.1	Microtus	arvalis	
AY708468.1	Microtus	arvalis	
AY708469.1	Microtus	arvalis	
FJ790003.1	Microtus	arvalis	
FJ790004.1	Microtus	arvalis	
AY708487.1	Microtus	arvalis	
AY708486.1	Microtus	arvalis	
FJ790005.1	Microtus	arvalis	

FJ790006.1	Microtus	arvalis	
FJ790007.1	Microtus	arvalis	
FJ790008.1	Microtus	arvalis	
FJ790009.1	Microtus	arvalis	
FJ790010.1	Microtus	arvalis	
AY708481.1	Microtus	arvalis	
FJ790011.1	Microtus	arvalis	
AY708517.1	Microtus	arvalis	
AY708518.1	Microtus	arvalis	
AY708470.1	Microtus	arvalis	
AY708471.1	Microtus	arvalis	
AY708472.1	Microtus	arvalis	
AY708473.1	Microtus	arvalis	
AY708505.1	Microtus	arvalis	
FJ790013.1	Microtus	arvalis	
AY708489.1	Microtus	arvalis	
AY708490.1	Microtus	arvalis	
AY708491.1	Microtus	arvalis	
AY708498.1	Microtus	arvalis	
AY220776.1	Microtus	arvalis	
AY708492.1	Microtus	arvalis	
AY708493.1	Microtus	arvalis	
AY708507.1	Microtus	arvalis	
FJ790014.1	Microtus	arvalis	
FJ790015.1	Microtus	arvalis	
FJ790016.1	Microtus	arvalis	
FJ790017.1	Microtus	arvalis	
FJ790018.1	Microtus	arvalis	
AY708474.1	Microtus	arvalis	
AY708475.1	Microtus	arvalis	
AY708476.1	Microtus	arvalis	
AY708477.1	Microtus	arvalis	
AY708478.1	Microtus	arvalis	
AY708479.1	Microtus	arvalis	
AY708480.1	Microtus	arvalis	
AY708494.1	Microtus	arvalis	
AY708520.1	Microtus	arvalis	
AY708495.1	Microtus	arvalis	
AY708496.1	Microtus	arvalis	
FJ790019.1	Microtus	arvalis	
FJ790020.1	Microtus	arvalis	
GU187381.1	Microtus	arvalis	
GU187382.1	Microtus	arvalis	
GU187383.1	Microtus	arvalis	

GU187384.1	Microtus	arvalis	
GU187385.1	Microtus	arvalis	
GU187386.1	Microtus	arvalis	
GU187362.1	Microtus	arvalis	
AY708499.1	Microtus	arvalis	
AY708500.1	Microtus	arvalis	
AY708523.1	Microtus	arvalis	
AY708524.1	Microtus	arvalis	
AY708502.1	Microtus	arvalis	
AY708501.1	Microtus	arvalis	
AY708525.1	Microtus	arvalis	
AY708497.1	Microtus	arvalis	
AY708503.1	Microtus	arvalis	
AY708484.1	Microtus	arvalis	
AY708485.1	Microtus	arvalis	
AY220770.1	Microtus	arvalis	
AY708483.1	Microtus	arvalis	
AY708511.1	Microtus	arvalis	
AY708488.1	Microtus	arvalis	
AY220787.1	Microtus	arvalis	
AY708521.1	Microtus	arvalis	
AY708504.1	Microtus	arvalis	
FJ790023.1	Microtus	arvalis	
AY708522.1	Microtus	arvalis	
AY708506.1	Microtus	arvalis	
AY220760.1	Microtus	arvalis	
AY220777.1	Microtus	arvalis	
EU439454.1	Microtus	arvalis	
EU439455.1	Microtus	arvalis	
EU439456.1	Microtus	arvalis	
EU439457.1	Microtus	arvalis	
EU439458.1	Microtus	arvalis	
EU439459.1	Microtus	arvalis	
AY220769.1	Microtus	arvalis	
FJ790026.1	Microtus	arvalis	
AY220766.1	Microtus	arvalis	
FJ790027.1	Microtus	arvalis	
FJ790028.1	Microtus	arvalis	
FJ790029.1	Microtus	arvalis	
FJ790030.1	Microtus	arvalis	
AY220778.1	Microtus	arvalis	
AY220779.1	Microtus	arvalis	
AY220780.1	Microtus	arvalis	
AY220781.1	Microtus	arvalis	

AY220782.1	Microtus	arvalis	
AY220783.1	Microtus	arvalis	
AY220785.1	Microtus	arvalis	
AY220786.1	Microtus	arvalis	
AY220773.1	Microtus	arvalis	
AY220772.1	Microtus	arvalis	
AY220774.1	Microtus	arvalis	
AY220775.1	Microtus	arvalis	
FJ790031.1	Microtus	arvalis	
FJ790032.1	Microtus	arvalis	
FJ790033.1	Microtus	arvalis	
AY220771.1	Microtus	arvalis	
AY220763.1	Microtus	arvalis	
AY220764.1	Microtus	arvalis	
AY220765.1	Microtus	arvalis	
AY220767.1	Microtus	arvalis	
AY220768.1	Microtus	arvalis	
AY220788.1	Microtus	arvalis	
AY220789.1	Microtus	arvalis	
AY220762.1	Microtus	arvalis	
GU954315.1	Microtus	arvalis	
GU954314.1	Microtus	arvalis	
EU439953.1	Microtus	arvalis	
EU439954.1	Microtus	arvalis	
EU439955.1	Microtus	arvalis	
EU439957.1	Microtus	arvalis	
EU439958.1	Microtus	arvalis	
EU439959.1	Microtus	arvalis	
AM991024.1	Microtus	arvalis	
AM991031.1	Microtus	arvalis	
AM991070.1	Microtus	arvalis	
AM991071.1	Microtus	arvalis	
AM991072.1	Microtus	arvalis	
AM991073.1	Microtus	arvalis	
AM991074.1	Microtus	arvalis	
AM991075.1	Microtus	arvalis	
AM991077.1	Microtus	arvalis	
AM991078.1	Microtus	arvalis	
AM991079.1	Microtus	arvalis	
AM991080.1	Microtus	arvalis	
AM991032.1	Microtus	arvalis	
AM991081.1	Microtus	arvalis	
AM991082.1	Microtus	arvalis	
AM991083.1	Microtus	arvalis	

AM991084.1	Microtus	arvalis	
AM991085.1	Microtus	arvalis	
AM991086.1	Microtus	arvalis	
AM991087.1	Microtus	arvalis	
AM991088.1	Microtus	arvalis	
AM991089.1	Microtus	arvalis	
AM991090.1	Microtus	arvalis	
AM991033.1	Microtus	arvalis	
AM991091.1	Microtus	arvalis	
AM991093.1	Microtus	arvalis	
AM991094.1	Microtus	arvalis	
AM991034.1	Microtus	arvalis	
AM991035.1	Microtus	arvalis	
AM991036.1	Microtus	arvalis	
AM991095.1	Microtus	arvalis	
AM991096.1	Microtus	arvalis	
AM991025.1	Microtus	arvalis	
AM991037.1	Microtus	arvalis	
AM991038.1	Microtus	arvalis	
AM991039.1	Microtus	arvalis	
AM991040.1	Microtus	arvalis	
AM991041.1	Microtus	arvalis	
AM991042.1	Microtus	arvalis	
AM991043.1	Microtus	arvalis	
AM991044.1	Microtus	arvalis	
AM991045.1	Microtus	arvalis	
AM991046.1	Microtus	arvalis	
AM991047.1	Microtus	arvalis	
AM991026.1	Microtus	arvalis	
AM991048.1	Microtus	arvalis	
AM991049.1	Microtus	arvalis	
AM991050.1	Microtus	arvalis	
AM991051.1	Microtus	arvalis	
AM991052.1	Microtus	arvalis	
AM991053.1	Microtus	arvalis	
AM991097.1	Microtus	arvalis	
AM991098.1	Microtus	arvalis	
AM991054.1	Microtus	arvalis	
AM991027.1	Microtus	arvalis	
AM991055.1	Microtus	arvalis	
AM991056.1	Microtus	arvalis	
AM991057.1	Microtus	arvalis	
AM991058.1	Microtus	arvalis	
AM991028.1	Microtus	arvalis	

AM991059.1	Microtus	arvalis	
AM991060.1	Microtus	arvalis	
AM991029.1	Microtus	arvalis	
AM991061.1	Microtus	arvalis	
AM991062.1	Microtus	arvalis	
AM991063.1	Microtus	arvalis	
AM991064.1	Microtus	arvalis	
AM991065.1	Microtus	arvalis	
AM991030.1	Microtus	arvalis	
AM991066.1	Microtus	arvalis	
AM991067.1	Microtus	arvalis	
AM991068.1	Microtus	arvalis	
AM991069.1	Microtus	arvalis	
DQ768134.1	Microtus	arvalis	
DQ768138.1	Microtus	arvalis	
DQ768133.1	Microtus	arvalis	
DQ768142.1	Microtus	arvalis	
DQ768131.1	Microtus	arvalis	
DQ768136.1	Microtus	arvalis	
DQ663660.1	Microtus	cabrerae	
AY513788.1	Microtus	cabrerae	
AY513789.1	Microtus	cabrerae	
GU954322.1	Microtus	cabrerae	
AY513796.1	Microtus	duodecimcostatus	
AY513797.1	Microtus	duodecimcostatus	
AJ717744.1	Microtus	duodecimcostatus	
AM392375.1	Microtus	duodecimcostatus	
AY513799.1	Microtus	gerbei	
AY513800.1	Microtus	gerbei	
AY513801.1	Microtus	gerbei	
AY513802.1	Microtus	gerbei	
AY513812.1	Microtus	lusitanicus	
AY513813.1	Microtus	lusitanicus	
AJ717746.1	Microtus	lusitanicus	
DQ663663.2	Microtus	multiplex	
AY513815.1	Microtus	multiplex	
AY513816.1	Microtus	multiplex	
AY513817.1	Microtus	multiplex	
AY513818.1	Microtus	multiplex	
AJ717747.1	Microtus	multiplex	
AY513824.1	Microtus	savii	
AY513825.1	Microtus	savii	
AY513826.1	Microtus	savii	
AY513827.1	Microtus	savii	

AY513828.1	Microtus	savii	
EU158792.1	Microtus	savii	
EU158800.1	Microtus	savii	
EU158780.1	Microtus	savii	
EU158781.1	Microtus	savii	
EU158782.1	Microtus	savii	
EU158790.1	Microtus	savii	
EU158791.1	Microtus	savii	
EU158788.1	Microtus	savii	
EU158776.1	Microtus	savii	
EU158785.1	Microtus	savii	
EU158786.1	Microtus	savii	
EU158789.1	Microtus	savii	
EU158778.1	Microtus	savii	
EU158796.1	Microtus	savii	
EU158787.1	Microtus	savii	
EU158783.1	Microtus	savii	
EU158784.1	Microtus	savii	
EU158804.1	Microtus	savii	
EU158803.1	Microtus	savii	
EU158802.1	Microtus	savii	
EU158794.1	Microtus	savii	
EU158795.1	Microtus	savii	
EU158777.1	Microtus	savii	
EU158793.1	Microtus	savii	
EU158797.1	Microtus	savii	
EU158798.1	Microtus	savii	
EU158801.1	Microtus	savii	
EU158799.1	Microtus	savii	
DQ663665.1	Microtus	subterraneus	
AY513832.1	Microtus	subterraneus	
AY513833.1	Microtus	subterraneus	
AY513834.1	Microtus	subterraneus	
AY513835.1	Microtus	subterraneus	
AY513836.1	Microtus	subterraneus	
AJ717745.1	Microtus	subterraneus	
AK131583.1	Mus	musculus	
AK131591.1	Mus	musculus	
AK131866.1	Mus	musculus	
EU349766.1	Mus	musculus	
HQ270439.1	Mus	musculus	
HQ270440.1	Mus	musculus	
HQ270441.1	Mus	musculus	
HQ270442.1	Mus	musculus	

HQ270443.1	Mus	musculus	
HQ270444.1	Mus	musculus	
HQ270445.1	Mus	musculus	
HQ270446.1	Mus	musculus	
HQ270447.1	Mus	musculus	
HQ270448.1	Mus	musculus	
HQ270434.1	Mus	musculus	
HQ270449.1	Mus	musculus	
HQ270450.1	Mus	musculus	
HQ270451.1	Mus	musculus	
HQ270452.1	Mus	musculus	
HQ270453.1	Mus	musculus	
HQ270454.1	Mus	musculus	
HQ270455.1	Mus	musculus	
HQ270435.1	Mus	musculus	
HQ270436.1	Mus	musculus	
HQ270437.1	Mus	musculus	
HQ270438.1	Mus	musculus	
AY057807.1	Mus	musculus	
AF520636.1	Mus	musculus	domesticus
AB125774.1	Mus	musculus	
AF520620.1	Mus	musculus	
AF520621.1	Mus	musculus	
AF520622.1	Mus	musculus	
AF520623.1	Mus	musculus	
AF520624.1	Mus	musculus	
AF520625.1	Mus	musculus	
HM222709.1	Mus	musculus	
AF520626.1	Mus	musculus	
AB033699.1	Mus	musculus	
AY057804.1	Mus	musculus	
AF520627.1	Mus	musculus	musculus
AF520628.1	Mus	musculus	musculus
AF520629.1	Mus	musculus	musculus
AB205273.1	Mus	musculus	
AB205274.1	Mus	musculus	
AB205275.1	Mus	musculus	
AY057810.1	Mus	spretus	
AY224678.1	Mus	spretus	
AF159398.1	Mus	spretus	
AB033700.1	Mus	spretus	
EU544663.1	Myocastor	coypus	
FJ528598.1	Myodes	glareolus	
EF408074.1	Myodes	glareolus	

EF408072.1	Myodes	glareolus	
FJ881489.1	Myodes	glareolus	
FJ881490.1	Myodes	glareolus	
FJ881491.1	Myodes	glareolus	
FJ881492.1	Myodes	glareolus	
FJ881405.1	Myodes	glareolus	
FJ881406.1	Myodes	glareolus	
FJ881407.1	Myodes	glareolus	
EF408073.1	Myodes	glareolus	
EU439963.1	Myodes	glareolus	
EU439964.1	Myodes	glareolus	
EU439965.1	Myodes	glareolus	
EU439966.1	Myodes	glareolus	
EU439967.1	Myodes	glareolus	
FJ881448.1	Myodes	glareolus	
FJ881449.1	Myodes	glareolus	
DQ090752.1	Myodes	glareolus	
DQ090761.1	Myodes	glareolus	
FJ881450.1	Myodes	glareolus	
FJ881451.1	Myodes	glareolus	
FJ881437.1	Myodes	glareolus	
FJ881438.1	Myodes	glareolus	
DQ090757.1	Myodes	glareolus	
DQ090758.1	Myodes	glareolus	
FJ881439.1	Myodes	glareolus	
FJ881453.1	Myodes	glareolus	
FJ881452.1	Myodes	glareolus	
FJ881454.1	Myodes	glareolus	
FJ881456.1	Myodes	glareolus	
FJ881455.1	Myodes	glareolus	
FJ881408.1	Myodes	glareolus	
FJ881409.1	Myodes	glareolus	
FJ881410.1	Myodes	glareolus	
FJ881411.1	Myodes	glareolus	
FJ881440.1	Myodes	glareolus	
FJ881441.1	Myodes	glareolus	
FJ881442.1	Myodes	glareolus	
FJ881443.1	Myodes	glareolus	
FJ881389.1	Myodes	glareolus	
FJ881390.1	Myodes	glareolus	
FJ881391.1	Myodes	glareolus	
FJ881392.1	Myodes	glareolus	
FJ881393.1	Myodes	glareolus	
FJ881412.1	Myodes	glareolus	

FJ881413.1	Myodes	glareolus	
FJ881414.1	Myodes	glareolus	
FJ881394.1	Myodes	glareolus	
FJ881395.1	Myodes	glareolus	
FJ881396.1	Myodes	glareolus	
FJ881397.1	Myodes	glareolus	
FJ881457.1	Myodes	glareolus	
FJ881458.1	Myodes	glareolus	
FJ881459.1	Myodes	glareolus	
FJ881460.1	Myodes	glareolus	
FJ881461.1	Myodes	glareolus	
FJ881415.1	Myodes	glareolus	
FJ881416.1	Myodes	glareolus	
FJ881417.1	Myodes	glareolus	
FJ881418.1	Myodes	glareolus	
FJ881419.1	Myodes	glareolus	
FJ881462.1	Myodes	glareolus	
FJ881463.1	Myodes	glareolus	
FJ881464.1	Myodes	glareolus	
DQ090751.1	Myodes	glareolus	
FJ881465.1	Myodes	glareolus	
FJ881466.1	Myodes	glareolus	
DQ090760.1	Myodes	glareolus	
FJ881467.1	Myodes	glareolus	
FJ881475.1	Myodes	glareolus	
FJ881476.1	Myodes	glareolus	
FJ881477.1	Myodes	glareolus	
FJ881478.1	Myodes	glareolus	
FJ881468.1	Myodes	glareolus	
FJ881469.1	Myodes	glareolus	
DQ090755.1	Myodes	glareolus	
FJ881470.1	Myodes	glareolus	
FJ881444.1	Myodes	glareolus	
FJ881445.1	Myodes	glareolus	
FJ881446.1	Myodes	glareolus	
FJ881447.1	Myodes	glareolus	
FJ881479.1	Myodes	glareolus	
FJ881480.1	Myodes	glareolus	
DQ090753.1	Myodes	glareolus	
EF378948.1	Myodes	glareolus	
FJ881402.1	Myodes	glareolus	
FJ881403.1	Myodes	glareolus	
FJ881398.1	Myodes	glareolus	
FJ881399.1	Myodes	glareolus	

FJ881400.1	Myodes	glareolus	
FJ881401.1	Myodes	glareolus	
DQ090759.1	Myodes	glareolus	
FJ881404.1	Myodes	glareolus	
FJ881420.1	Myodes	glareolus	
FJ881421.1	Myodes	glareolus	
FJ881424.1	Myodes	glareolus	
FJ881422.1	Myodes	glareolus	
FJ881423.1	Myodes	glareolus	
FJ881425.1	Myodes	glareolus	
FJ881426.1	Myodes	glareolus	
FJ881427.1	Myodes	glareolus	
FJ881428.1	Myodes	glareolus	
FJ881429.1	Myodes	glareolus	
FJ881430.1	Myodes	glareolus	
FJ881471.1	Myodes	glareolus	
FJ881472.1	Myodes	glareolus	
FJ881473.1	Myodes	glareolus	
FJ881474.1	Myodes	glareolus	
EF408060.1	Myodes	glareolus	
FJ881481.1	Myodes	glareolus	
FJ881482.1	Myodes	glareolus	
FJ881483.1	Myodes	glareolus	
FJ881484.1	Myodes	glareolus	
FJ881485.1	Myodes	glareolus	
FJ881486.1	Myodes	glareolus	
FJ881487.1	Myodes	glareolus	
FJ881488.1	Myodes	glareolus	
FJ881431.1	Myodes	glareolus	
FJ881432.1	Myodes	glareolus	
FJ881433.1	Myodes	glareolus	
FJ881434.1	Myodes	glareolus	
FJ881435.1	Myodes	glareolus	
FJ881436.1	Myodes	glareolus	
DQ090756.1	Myodes	glareolus	
EU232136.1	Myodes	glareolus	
EU232146.1	Myodes	glareolus	
EU232147.1	Myodes	glareolus	
EU232148.1	Myodes	glareolus	
EU232150.1	Myodes	glareolus	
EU232151.1	Myodes	glareolus	
EU232152.1	Myodes	glareolus	
EU232153.1	Myodes	glareolus	
EU232154.1	Myodes	glareolus	

EU232155.1	Myodes	glareolus	
EU232137.1	Myodes	glareolus	
EU232156.1	Myodes	glareolus	
EU232157.1	Myodes	glareolus	
EU232158.1	Myodes	glareolus	
EU232159.1	Myodes	glareolus	
EU232160.1	Myodes	glareolus	
EU232161.1	Myodes	glareolus	
EU232162.1	Myodes	glareolus	
EU232164.1	Myodes	glareolus	
EU232165.1	Myodes	glareolus	
EU232166.1	Myodes	glareolus	
EU232167.1	Myodes	glareolus	
EU232168.1	Myodes	glareolus	
EU232169.1	Myodes	glareolus	
EU232170.1	Myodes	glareolus	
EU232171.1	Myodes	glareolus	
EU232139.1	Myodes	glareolus	
EU232140.1	Myodes	glareolus	
EU232141.1	Myodes	glareolus	
EU232142.1	Myodes	glareolus	
EU232144.1	Myodes	glareolus	
EF408066.1	Myodes	glareolus	
EU077269.1	Myodes	glareolus	
EU035707.1	Myodes	glareolus	
EU035639.1	Myodes	glareolus	
EU035648.1	Myodes	glareolus	
EU035649.1	Myodes	glareolus	
EU035650.1	Myodes	glareolus	
EU035651.1	Myodes	glareolus	
EU035652.1	Myodes	glareolus	
EU035653.1	Myodes	glareolus	
EU035654.1	Myodes	glareolus	
EU035656.1	Myodes	glareolus	
EU035708.1	Myodes	glareolus	
EU035640.1	Myodes	glareolus	
EU035657.1	Myodes	glareolus	
EU035658.1	Myodes	glareolus	
EU035659.1	Myodes	glareolus	
EU035665.1	Myodes	glareolus	
EU035666.1	Myodes	glareolus	
EU035709.1	Myodes	glareolus	
EU035641.1	Myodes	glareolus	
EU035667.1	Myodes	glareolus	

EU035668.1	Myodes	glareolus	
EU035669.1	Myodes	glareolus	
EU035670.1	Myodes	glareolus	
EU035671.1	Myodes	glareolus	
EU035672.1	Myodes	glareolus	
EU035673.1	Myodes	glareolus	
EU035674.1	Myodes	glareolus	
EU035675.1	Myodes	glareolus	
EU035642.1	Myodes	glareolus	
EU035710.1	Myodes	glareolus	
EU035680.1	Myodes	glareolus	
EU035681.1	Myodes	glareolus	
EU035682.1	Myodes	glareolus	
EU035683.1	Myodes	glareolus	
EU035684.1	Myodes	glareolus	
EU035685.1	Myodes	glareolus	
EU035686.1	Myodes	glareolus	
EU035643.1	Myodes	glareolus	
EU035687.1	Myodes	glareolus	
EU035688.1	Myodes	glareolus	
EU035689.1	Myodes	glareolus	
EU035690.1	Myodes	glareolus	
EU035691.1	Myodes	glareolus	
EU035692.1	Myodes	glareolus	
EU035693.1	Myodes	glareolus	
EU035644.1	Myodes	glareolus	
EU035697.1	Myodes	glareolus	
EU035699.1	Myodes	glareolus	
EU035700.1	Myodes	glareolus	
EU035701.1	Myodes	glareolus	
EU035702.1	Myodes	glareolus	
EU035703.1	Myodes	glareolus	
EU035704.1	Myodes	glareolus	
EU035705.1	Myodes	glareolus	
EU035706.1	Myodes	glareolus	
EU035645.1	Myodes	glareolus	
EU035646.1	Myodes	glareolus	
GQ339501.1	Myodes	glareolus	
EU523549.1	Myodes	glareolus	
EU523550.1	Myodes	glareolus	
EU523551.1	Myodes	glareolus	
EU523552.1	Myodes	glareolus	
EU523553.1	Myodes	glareolus	
GQ339500.1	Myodes	glareolus	

GQ339497.1	Myodes	glareolus	
GQ339499.1	Myodes	glareolus	
GQ339496.1	Myodes	glareolus	
GQ339502.1	Myodes	glareolus	
GQ339498.1	Myodes	glareolus	
GQ339503.1	Myodes	glareolus	
EF408068.1	Myodes	glareolus	
EF408061.1	Myodes	glareolus	
FJ640950.1	Myodes	glareolus	
FJ640951.1	Myodes	glareolus	
FJ640865.1	Myodes	glareolus	
FJ640866.1	Myodes	glareolus	
FJ640867.1	Myodes	glareolus	
FJ640868.1	Myodes	glareolus	
FJ640869.1	Myodes	glareolus	
FJ640870.1	Myodes	glareolus	
FJ640871.1	Myodes	glareolus	
FJ640872.1	Myodes	glareolus	
FJ640873.1	Myodes	glareolus	
FJ640874.1	Myodes	glareolus	
FJ640875.1	Myodes	glareolus	
FJ640876.1	Myodes	glareolus	
FJ640877.1	Myodes	glareolus	
FJ640878.1	Myodes	glareolus	
FJ640879.1	Myodes	glareolus	
FJ640880.1	Myodes	glareolus	
FJ640881.1	Myodes	glareolus	
FJ640882.1	Myodes	glareolus	
FJ640883.1	Myodes	glareolus	
FJ640884.1	Myodes	glareolus	
FJ640885.1	Myodes	glareolus	
FJ640886.1	Myodes	glareolus	
FJ640887.1	Myodes	glareolus	
FJ640888.1	Myodes	glareolus	
FJ640889.1	Myodes	glareolus	
FJ640890.1	Myodes	glareolus	
FJ640891.1	Myodes	glareolus	
FJ640892.1	Myodes	glareolus	
FJ640893.1	Myodes	glareolus	
FJ640894.1	Myodes	glareolus	
FJ640895.1	Myodes	glareolus	
FJ640896.1	Myodes	glareolus	
FJ640897.1	Myodes	glareolus	
FJ640898.1	Myodes	glareolus	

FJ640899.1	Myodes	glareolus	
FJ640900.1	Myodes	glareolus	
FJ640901.1	Myodes	glareolus	
FJ640902.1	Myodes	glareolus	
FJ640903.1	Myodes	glareolus	
FJ640904.1	Myodes	glareolus	
FJ640905.1	Myodes	glareolus	
FJ640906.1	Myodes	glareolus	
FJ640907.1	Myodes	glareolus	
FJ640908.1	Myodes	glareolus	
FJ640909.1	Myodes	glareolus	
FJ640910.1	Myodes	glareolus	
FJ640911.1	Myodes	glareolus	
FJ640912.1	Myodes	glareolus	
FJ640913.1	Myodes	glareolus	
FJ640914.1	Myodes	glareolus	
FJ640915.1	Myodes	glareolus	
FJ640916.1	Myodes	glareolus	
FJ640917.1	Myodes	glareolus	
FJ640918.1	Myodes	glareolus	
FJ640919.1	Myodes	glareolus	
FJ640920.1	Myodes	glareolus	
FJ640921.1	Myodes	glareolus	
FJ640922.1	Myodes	glareolus	
FJ640923.1	Myodes	glareolus	
FJ640924.1	Myodes	glareolus	
FJ640925.1	Myodes	glareolus	
FJ640926.1	Myodes	glareolus	
FJ640927.1	Myodes	glareolus	
FJ640928.1	Myodes	glareolus	
FJ640929.1	Myodes	glareolus	
FJ640930.1	Myodes	glareolus	
FJ640931.1	Myodes	glareolus	
FJ640932.1	Myodes	glareolus	
FJ640933.1	Myodes	glareolus	
FJ640934.1	Myodes	glareolus	
FJ640935.1	Myodes	glareolus	
FJ640936.1	Myodes	glareolus	
FJ640937.1	Myodes	glareolus	
FJ640938.1	Myodes	glareolus	
FJ640939.1	Myodes	glareolus	
FJ640940.1	Myodes	glareolus	
FJ640941.1	Myodes	glareolus	
FJ640942.1	Myodes	glareolus	

FJ640943.1	Myodes	glareolus	
FJ640944.1	Myodes	glareolus	
FJ640945.1	Myodes	glareolus	
FJ640946.1	Myodes	glareolus	
FJ640947.1	Myodes	glareolus	
FJ640952.1	Myodes	glareolus	
FJ640948.1	Myodes	glareolus	
FJ640953.1	Myodes	glareolus	
FJ640949.1	Myodes	glareolus	
EU483563.1	Myodes	glareolus	
EU483594.1	Myodes	glareolus	
EU483547.1	Myodes	glareolus	
EU483593.1	Myodes	glareolus	
EU483546.1	Myodes	glareolus	
EU483553.1	Myodes	glareolus	
EU483551.1	Myodes	glareolus	
EU483552.1	Myodes	glareolus	
EU483592.1	Myodes	glareolus	
EU483591.1	Myodes	glareolus	
EU483590.1	Myodes	glareolus	
EU483589.1	Myodes	glareolus	
EU483588.1	Myodes	glareolus	
EU483587.1	Myodes	glareolus	
EU483576.1	Myodes	glareolus	
EU483575.1	Myodes	glareolus	
EU483574.1	Myodes	glareolus	
EU483577.1	Myodes	glareolus	
EU483578.1	Myodes	glareolus	
EU483535.1	Myodes	glareolus	
EU483536.1	Myodes	glareolus	
EU483557.1	Myodes	glareolus	
EU483558.1	Myodes	glareolus	
EU483537.1	Myodes	glareolus	
EU483538.1	Myodes	glareolus	
EF408078.1	Myodes	glareolus	
EF408076.1	Myodes	glareolus	
EF408080.1	Myodes	glareolus	
EF408079.1	Myodes	glareolus	
EU483541.1	Myodes	glareolus	
EU483542.1	Myodes	glareolus	
EF408082.1	Myodes	glareolus	
EU483579.1	Myodes	glareolus	
EF408083.1	Myodes	glareolus	
EU483545.1	Myodes	glareolus	

EU483519.1	Myodes	glareolus	
EU483556.1	Myodes	glareolus	
EU483540.1	Myodes	glareolus	
EU483518.1	Myodes	glareolus	
EF408069.1	Myodes	glareolus	
EF408071.1	Myodes	glareolus	
EU483586.1	Myodes	glareolus	
EU483530.1	Myodes	glareolus	
EU483534.1	Myodes	glareolus	
EU483532.1	Myodes	glareolus	
EU483533.1	Myodes	glareolus	
EU483585.1	Myodes	glareolus	
EU483584.1	Myodes	glareolus	
EU483583.1	Myodes	glareolus	
EU483582.1	Myodes	glareolus	
EU483581.1	Myodes	glareolus	
EU483580.1	Myodes	glareolus	
EU483570.1	Myodes	glareolus	
EU483569.1	Myodes	glareolus	
EU483568.1	Myodes	glareolus	
EU483561.1	Myodes	glareolus	
EU483571.1	Myodes	glareolus	
EU483567.1	Myodes	glareolus	
EU483566.1	Myodes	glareolus	
EU483565.1	Myodes	glareolus	
EU483520.1	Myodes	glareolus	
EU483521.1	Myodes	glareolus	
EU483522.1	Myodes	glareolus	
EU483523.1	Myodes	glareolus	
EU483525.1	Myodes	glareolus	
EU483529.1	Myodes	glareolus	
EU483528.1	Myodes	glareolus	
EU483526.1	Myodes	glareolus	
EU483527.1	Myodes	glareolus	
EU483554.1	Myodes	glareolus	
EU483555.1	Myodes	glareolus	
EU483548.1	Myodes	glareolus	
EU483549.1	Myodes	glareolus	
EU483550.1	Myodes	glareolus	
DQ768151.1	Myodes	glareolus	
EU349782.1	Rattus	norvegicus	
GU592980.1	Rattus	norvegicus	
GU592954.1	Rattus	norvegicus	
GU592955.1	Rattus	norvegicus	

GU592956.1	Rattus	norvegicus	
GU592957.1	Rattus	norvegicus	
GU592958.1	Rattus	norvegicus	
GU592959.1	Rattus	norvegicus	
GU592960.1	Rattus	norvegicus	
GU592961.1	Rattus	norvegicus	
GU592962.1	Rattus	norvegicus	
GU592963.1	Rattus	norvegicus	
GU592964.1	Rattus	norvegicus	
GU592966.1	Rattus	norvegicus	
GU592967.1	Rattus	norvegicus	
GU592968.1	Rattus	norvegicus	
GU592979.1	Rattus	norvegicus	
GU592969.1	Rattus	norvegicus	
GU592970.1	Rattus	norvegicus	
GU592971.1	Rattus	norvegicus	
GU592975.1	Rattus	norvegicus	
GU592976.1	Rattus	norvegicus	
GU592977.1	Rattus	norvegicus	
GU592982.1	Rattus	norvegicus	
GU592983.1	Rattus	norvegicus	
GU592984.1	Rattus	norvegicus	
GU592985.1	Rattus	norvegicus	
HM222710.1	Rattus	norvegicus	
GU592986.1	Rattus	norvegicus	
GU592987.1	Rattus	norvegicus	
GU592988.1	Rattus	norvegicus	
GU592978.1	Rattus	norvegicus	
GU592989.1	Rattus	norvegicus	
GU592991.1	Rattus	norvegicus	
GU592992.1	Rattus	norvegicus	
GU592972.1	Rattus	norvegicus	
GU592973.1	Rattus	norvegicus	
GU592974.1	Rattus	norvegicus	
GU592993.1	Rattus	norvegicus	
GU592994.1	Rattus	norvegicus	
GU592981.1	Rattus	norvegicus	
GU592995.1	Rattus	norvegicus	
GU592990.1	Rattus	norvegicus	
GU592965.1	Rattus	norvegicus	
GU592996.1	Rattus	norvegicus	
GU592997.1	Rattus	norvegicus	
AB355902.1	Rattus	norvegicus	
AB355903.1	Rattus	norvegicus	

AB033713.1	Rattus	norvegicus	
HM217370.1	Rattus	norvegicus	
HM217429.1	Rattus	norvegicus	
HM217481.1	Rattus	norvegicus	
AB211039.1	Rattus	rattus	
AB033702.1	Rattus	rattus	
HM217368.1	Rattus	rattus	
HM217366.1	Rattus	rattus	
HM217367.1	Rattus	rattus	
AB292679.1	Sciurus	vulgaris	
AB292680.1	Sciurus	vulgaris	
AB292681.1	Sciurus	vulgaris	
AB030028.1	Sciurus	vulgaris	
AB030027.1	Sciurus	vulgaris	
AB030026.1	Sciurus	vulgaris	
AB015083.1	Sus	scrofa	
AB015082.1	Sus	scrofa	
AB015078.1	Sus	scrofa	
AB015075.1	Sus	scrofa	
AB015074.1	Sus	scrofa	
AB015070.1	Sus	scrofa	
AB015069.1	Sus	scrofa	
AB015068.1	Sus	scrofa	
AB015066.1	Sus	scrofa	
AM492566.2	Sus	scrofa	
AM492605.2	Sus	scrofa	
AM492628.2	Sus	scrofa	
AM492607.2	Sus	scrofa	
AM492606.1	Sus	scrofa	
AM492624.1	Sus	scrofa	
AM492623.1	Sus	scrofa	
AM492622.1	Sus	scrofa	
AM492621.1	Sus	scrofa	
AM492620.1	Sus	scrofa	
AM492619.1	Sus	scrofa	
D34635.1	Bos	taurus	
DQ186222.1	Bos	taurus	
DQ186213.1	Bos	taurus	
DQ186289.1	Bos	taurus	
DQ186283.1	Bos	taurus	
DQ186279.1	Bos	taurus	
DQ186265.1	Bos	taurus	
DQ186263.1	Bos	taurus	
DQ186260.1	Bos	taurus	

DQ186259.1	Bos	taurus	
DQ186254.1	Bos	taurus	
DQ186253.1	Bos	taurus	
DQ186252.1	Bos	taurus	
DQ186245.1	Bos	taurus	
DQ186242.1	Bos	taurus	
DQ186233.1	Bos	taurus	
DQ186228.1	Bos	taurus	
DQ186212.1	Bos	taurus	
DQ186211.1	Bos	taurus	
DQ186209.1	Bos	taurus	
D84205.1	Ovis	aries	
AB006800.1	Ovis	aries	
D84201.1	Capra	hircus	
AB004075.1	Capra	hircus	
AB004074.1	Capra	hircus	
AB004073.1	Capra	hircus	
AB004071.1	Capra	hircus	
AB004070.1	Capra	hircus	
AB004072.1	Capra	hircus	
DQ093614.1	Capra	hircus	
AB110597.1	Capra	hircus	
AB110596.1	Capra	hircus	
DQ073048.1	Capra	hircus	
FJ556564.1	Capra	hircus	
AB736144.1	Capra	hircus	
AB736131.1	Capra	hircus	
EU130775.1	Capra	hircus	
DQ514546.1	Capra	hircus	
DQ089480.1	Capra	hircus	
DQ089476.1	Capra	hircus	
AB044986.1	Gallus	gallus	
AB044987.1	Gallus	gallus	
AB044985.1	Gallus	gallus	
AF153499.1	Gallus	gallus	
AF354171.1	Gallus	gallus	
AF195631.1	Gallus	gallus	
AF195630.1	Gallus	gallus	
AF195629.1	Gallus	gallus	
AF195628.1	Gallus	gallus	
AF028795.1	Gallus	gallus	
AB044984.1	Gallus	gallus	
AY029583.1	Gallus	gallus	
AY029582.1	Gallus	gallus	

AF119093.1	Gallus	gallus	
AF102874.1	Gallus	gallus	
AF362406.1	Gallus	gallus	
AY026059.1	Gallus	gallus	
>GQ120440.1	Anser	anser	
EU585613.1	Anser	anser	
AY552162.1	Anser	cygnoides	
AY552165.1	Anser	cygnoides	
AY552164.1	Anser	cygnoides	
AY552163.1	Anser	cygnoides	
EU585616.1	Anser	cygnoides	
EU863202.1	Anser	cygnoides	
EU863201.1	Anser	cygnoides	
AY552162.1	Anser	cygnoides	
AY552165.1	Anser	cygnoides	
AY552164.1	Anser	cygnoides	
AY552163.1	Anser	cygnoides	
EU585616.1	Anser	cygnoides	
EU863202.1	Anser	cygnoides	
EU863201.1	Anser	cygnoides	
EU492281.1	Salmo	salar	
BT044011.1	Salmo	salar	
AF053591.1	Salmo	salar	
FJ435619.1	Salmo	salar	
EU492280.1	Salmo	salar	
EF584212.1	Salmo	salar	
FJ435620.1	Salmo	salar	
FJ435618.1	Salmo	salar	
AF202032.1	Salmo	salar	
AF059098.1	Cairina	moschata	
